# Spontanes Pneumomediastinum bei einer jungen Frau

**DOI:** 10.1007/s00101-026-01708-9

**Published:** 2026-07-07

**Authors:** André Thyroff, Katharina Müller-Peltzer, Joachim Bansbach, Johannes Kalbhenn

**Affiliations:** 1 Klinik für Anästhesiologie und Intensivmedizin, Universitätsklinikum Freiburg, Hugstetter Str. 55, 79106 Freiburg im Breisgau, Deutschland; 2 Klinik für Diagnostische und Interventionelle Radiologie, Universitätsklinikum Freiburg, Hugstetter Str. 55, 79106 Freiburg im Breisgau, Deutschland

## Falldarstellung

### Anamnese

Eine bisher gesunde 19-jährige Patientin stellte sich mit Dyspnoe seit dem Vortag und linksthorakalen atemabhängigen Schmerzen in unserem universitären Notfallzentrum vor.

Eine Woche zuvor habe ein leichter Atemwegsinfekt bestanden, und vor 3 Wochen habe sie angefangen, ihr eigenes Auto zu lackieren. Die Dyspnoe habe im Rahmen eines Hustenanfalls am Vortag begonnen. Weiterhin ließen sich Rauchen seit 2 Jahren (ca. 5 Zigaretten täglich) und regelmäßiges Dampfen von E‑Zigaretten („Vaping“) seit etwa einem Jahr eruieren.

### Befund

Bei Aufnahme betrug die Sauerstoffsättigung unter Raumluft 96 %. Die weiteren Vitalparameter waren bis auf eine Tachykardie (102/min) und eine erhöhte Atemfrequenz (20/min) unauffällig. Das 12-Kanal-EKG zeigte eine milde Sinustachykardie. Auskultatorisch fiel eine Bronchospastik auf.

Die Laborwerte waren abgesehen von einer Leukozytose mit Neutrophilie unauffällig (s. Tab. 1). Eine venöse Blutgasanalyse erbrachte keine relevanten Auffälligkeiten. Die Point-of-Care-PCR-Testung auf SARS-CoV‑2, Influenza A/B und RSV blieb negativ.

**Tab. 1:** Laborwerte bei Aufnahme (Auszug)

Parameter [Einheit]	Wert
Leukozyten [Tsd/µl]	*16,65 ↑*
Hämoglobin [g/dl]	13,8
Thrombozyten [Tsd/µl]	326
Neutrophile masch. [%]	*79,2 ↑*
Neutrophile masch. abs. [Tsd/µl]	*13,22 ↑*
D‑Dimere [mg/l] FEU	0,39
Troponin T [ng/l]	< 5,0

**Abb. 1:**
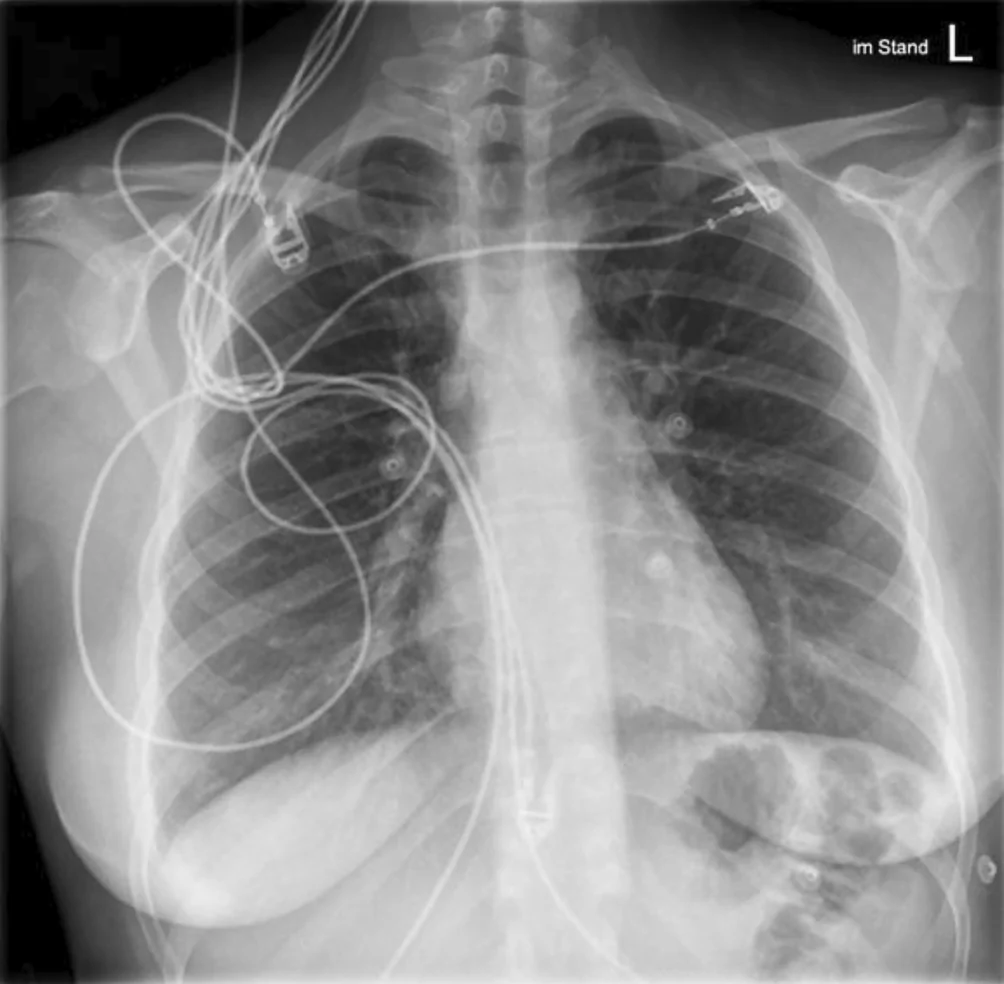
Röntgenaufnahme des Thorax

In der Röntgenaufnahme des Thorax (Abb. 1) waren diskret verschattete Randwinkel auffällig. Echokardiographisch zeigte sich eine gute biventrikuläre Pumpfunktion ohne regionale Wandbewegungsstörungen. Weitere Auffälligkeiten wie Perikarderguss, höhergradige Klappenvitien oder Rechtsherzbelastungszeichen konnten nicht erhoben werden. Die Sonographie des Abdomens war ebenfalls regelrecht.

### Therapie und Verlauf

**Abb. 2:**
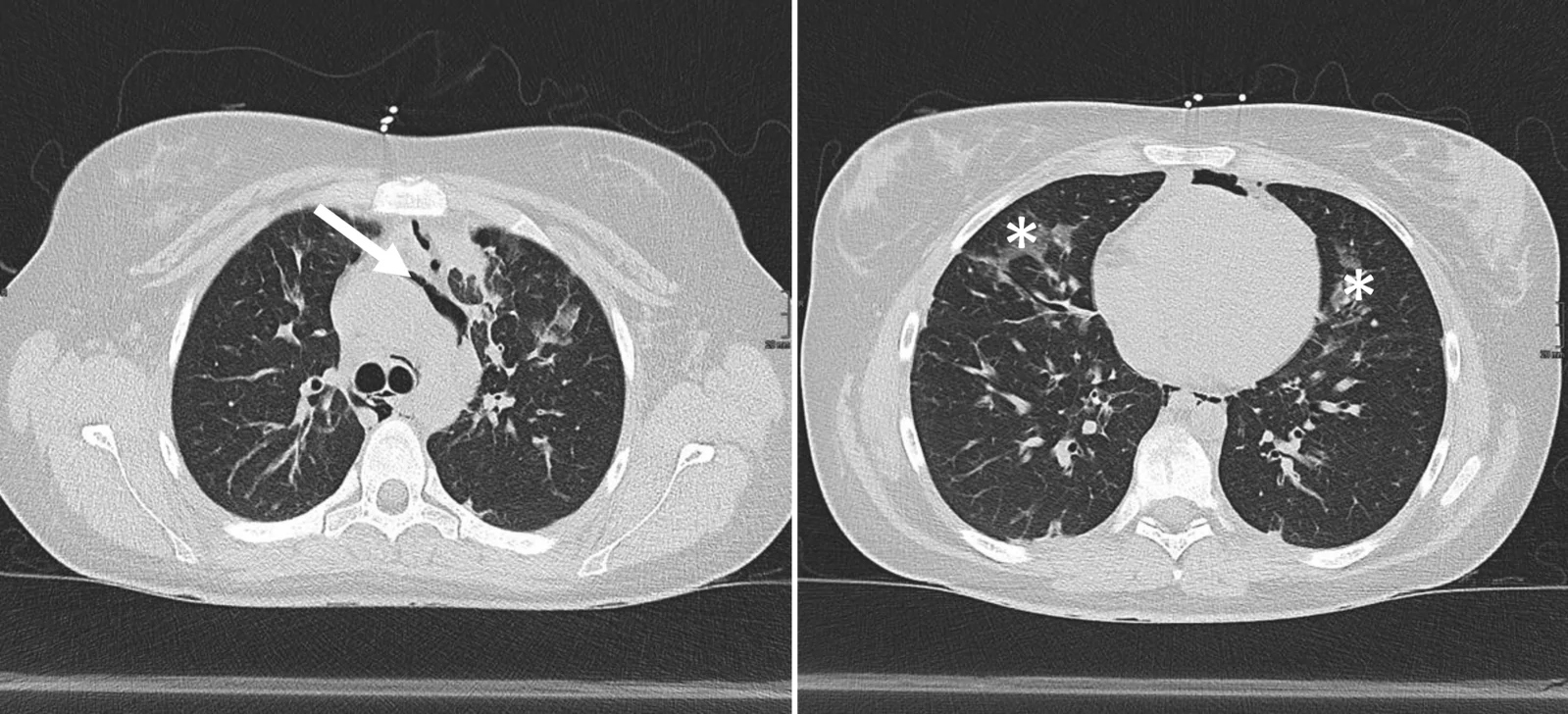
Kontrastmittelgestützte Computertomographie. *Pfeil*: Mediastinalemphysem. *Asterisken*: atypische Infiltrate

Trotz bronchodilatativer Maßnahmen sowie Gabe eines Glukokortikosteroids wurde zeitnah die Zufuhr von 2 l/min Sauerstoff notwendig. Diese musste über die kommenden Stunden sukzessive eskaliert werden. Bei gleichzeitig zunehmender Dysphagie wurde eine Computertomographie von Hals und Thorax (Abb. 2) veranlasst. Hier zeigten sich ein ausgedehntes retropharyngeales Emphysem von tiefzervikal bis auf Höhe des Nasopharynx, ein Mediastinalemphysem bis auf Höhe des thorakoabdominellen Übergangs sowie bilaterale atypische Infiltrate, teils mit Milchglaskonfiguration.

Bei subjektiv zunehmender Atemwegskompromittierung wurde die Patientin auf die anästhesiologische Intensivtherapiestation verlegt.

Bei Übernahme war sie wach, tachypnoeisch und erhielt 9 l/min Sauerstoff über eine Gesichtsmaske mit Reservoir. Stridor bestand nicht. Wegen der akuten hypoxischen respiratorischen Insuffizienz und emphysematischen Weichteilschwellung im Oropharynx wurde eine Atemwegssicherung als fiberoptische Intubation unter erhaltener Spontanatmung durchgeführt. Verletzungen des Tracheobronchialsystems, Ösophagus und Pharynx wurden im Anschluss bronchoskopisch und endoskopisch ausgeschlossen.

Die respiratorische Insuffizienz persistierte unter kontrollierter Beatmung und assistierter Spontanatmung (p_a_O_2_/F_i_O_2_-Quotient nach Intubation 93). Hierbei war eine Restriktion mit niedriger Compliance auffällig. Aufgrund einer möglicherweise durch kontinuierliche Applikation eines positiven endexspiratorischen Drucks aggravierten Gasaustauschstörung durch das Mediastinalemphysem erfolgte daher gleichentags die Extubation. Unter nasaler Hochflusssauerstofftherapie und intermittierender nichtinvasiver Beatmung war zunächst keine Verbesserung der Oxygenierung möglich.

### Diagnose

Der Procalcitoninwert war mit 0,06 ng/ml niedrig, sodass wir nicht von einer bakteriell-infektiösen Genese der Infiltrate ausgingen. In der Bronchoskopie war lediglich eine gerötete Schleimhaut auffällig. Eine bronchoalveoläre Lavage wurde nicht durchgeführt. Legionellen- und Pneumokokkenantigen im Urin waren negativ, ebenso wie die serologische Diagnostik auf HIV. Eine rheumatologische oder autoimmunologische Genese war bei Normwerten für Immunglobuline, ANA, ANCA, Anti-Phospholipid-Ak und Komplementfaktoren unwahrscheinlich. Retrospektiv war lediglich der Anti-Streptolysin-Titer seriell erhöht (548 und 534 IE/ml). Eine Eosinophilie lag im Differenzialblutbild nicht vor (Eosinophile 1,3 % bzw. 0,21 Tsd/µl). Fieber trat nur einmalig am zweiten Behandlungstag auf. Die biventrikuläre Pumpfunktion zeigte sich in wiederholten Untersuchungen regelrecht und ohne Zeichen einer Rechtsherzbelastung. Die Infobox gibt einen Überblick über wichtige Differenzialdiagnosen pulmonaler Milchglasinfiltrate.

#### Infobox Auswahl Differenzialdiagnosen bei pulmonalen Milchglasinfiltraten nach [1, 2]

- ARDS
- Eosinophile Pneumonie
- Exogen-allergische Alveolitis
- Pneumonie (virale/atypische) inklusive Pneumocystis-jirovecii-Pneumonie und COVID-19-Pneumonie
- Medikamenten-induzierter Lungenschaden (z. B. Amiodaron, Pembrolizumab)
- EVALI

Aufgrund der Anamnese erschien EVALI („E-cigarette or vaping product use-associated lung injury“) möglich, und wir begannen am dritten Behandlungstag eine Glukokortikoidstoßtherapie mit Methylprednisolon und Prednison über jeweils 2 Tage. Bei erneuter Bronchospastik wurde eine intravenöse Zufuhr von Reproterol begonnen und über 3 Tage ausgeschlichen. Die Lungenfunktion erholte sich sukzessive, sodass der p_a_O_2_/F_i_O_2_-Quotient ab dem fünften Tag über 200 und ab dem sechsten Tag über 300 lag. In der Röntgenuntersuchung des Thorax war das Mediastinalemphysem am dritten Behandlungstag bereits regredient. Elf Tage nach Aufnahme konnte die Patientin ohne Sauerstoffbedarf auf eine Normalstation verlegt werden.

## Diskussion

EVALI ist eine akute und teils schwer verlaufende Pneumonitis, welche durch toxische Inhaltsstoffe insbesondere in Zusammenhang mit illegalen Beimischungen von Liquids in E‑Zigaretten hervorgerufen wird [3]. Typisch sind neben einer schwerwiegenden Gasaustauschstörung auch Milchglasinfiltrate [4]. Die Gabe von Glukokortikoiden ist die aktuell gängige Therapie. EVALI trat erstmals im Rahmen einer größeren Häufung mit etlichen Todesfällen in den USA im Jahr 2019 auf [5]. Für Deutschland gibt es keine exakten Daten zu Inzidenz und Prävalenz, allerdings zuletzt immer wieder Fallberichte [6–8].

Im vorliegenden Fall ergab sich differenzialdiagnostisch weder ein Anhalt für eine infektiöse noch für eine rheumatologische, kardiale oder neoplastische Genese. Bedauerlicherweise wurde auf eine Immunobronchiallavage verzichtet. Eine eosinophile Pneumonie als weitere Differenzialdiagnose kommt auch bei unauffälliger Bronchoskopie infrage und kann ohne Zytologie retrospektiv nicht ausgeschlossen werden. Unsere Patientin gab überdies an, keine illegalen Vaping-Produkte, sondern lediglich in Deutschland legal erhältliche Pods eines Herstellers in verschiedenen Geschmacksrichtungen konsumiert zu haben. Prinzipiell wäre im EVALI-Verdachtsfall eine toxikologische Aufarbeitung des konsumierten Liquids, wie in anderen Fallberichten dargestellt, möglich gewesen [8]. Zusammenfassend sind hier trotz diagnostischer Unsicherheiten allerdings die Kriterien des Center for Disease Control and Prevention für EVALI [9] erfüllt:

- Hospitalisierung aufgrund respiratorischer Symptomatik,
- nach Gebrauch von E‑Zigaretten/Vaping/Dampfen in den vorangegangenen 90 Tagen und
- Milchglasinfiltrate in der Computertomographie ohne eindeutige andere Ätiologie,
- Ausschluss respiratorischer Infekte (u. a. SARS-CoV‑2, Influenza, S. pneumoniae, Legionellen, ggf. HIV-assoziierte opportunistische Infektionen) mittels PCR/Blutkultur/Urinantigen, ggf. Sputumkultur bei produktivem Husten, ggf. BAL,
- kein Anhalt für kardiale, rheumatologische oder neoplastische Erkrankung.

Weiterhin ist Vaping, ebenso wie Zigarettenrauchen, mit Spontanpneumothoraces assoziiert. Ursächlich hierfür scheinen entzündliche alveoläre Prozesse und die Bildung von Bullae zu sein [10]. Das Auftreten eines Pneumomediastinums ist ebenfalls bereits im Rahmen von EVALI beschrieben worden [11, 12]. Hierfür kommt am ehesten der Macklin-Effekt mit Übertritt von Luft über das peribronchovaskuläre Interstitium nach Alveolarruptur infrage. Ein spontanes Pneumomediastinum durch starke intrathorakale Druckerhöhung ist als Hamman-Syndrom beschrieben [13]. Der kürzlich durchgemachte respiratorische Infekt sowie die stattgehabte Exposition gegenüber Lacken mag ein Trigger für starkes Husten mit konsekutivem Pneumomediastinum gewesen sein.

Klinisch sind hier gleichzeitig auch die Kriterien eines ARDS sowohl nach Berlin-Definition als auch nach New Global Definition wegen der unter nasaler Hochflusssauerstofftherapie fortbestehenden respiratorischen Insuffizienz erfüllt [14].

## Fazit für die Praxis

Retrospektiv erscheint für uns EVALI aus klinischer Sicht die wahrscheinlichste Ursache für das spontane Pneumomediastinum bei dieser jungen Patientin zu sein. Da es sich um eine Ausschlussdiagnose handelt, möchten wir dafür sensibilisieren, bei entsprechender Anamnese, respiratorischer Insuffizienz, bilateralen Infiltraten oder bei spontanem Pneumothorax oder Pneumomediastinum, diese Entität frühzeitig in die differenzialdiagnostischen Überlegungen einzubeziehen. Zur Abgrenzung wichtiger Differenzialdiagnosen würden wir grundsätzlich zur Durchführung einer bronchoalveolären Lavage mit zytologischer Differenzierung raten. Analog zum ARDS empfehlen wir eine lungenprotektive Respiratorunterstützung, die in unserem Fall überwiegend mit nasaler Hochflusssauerstofftherapie durchgeführt wurde.

## Data Availability

Alle dieser Arbeit zugrunde liegenden Daten sind in diesem Artikel enthalten.
